# Human Specific Regulation of the Telomerase Reverse Transcriptase Gene

**DOI:** 10.3390/genes7070030

**Published:** 2016-06-28

**Authors:** Fan Zhang, De Cheng, Shuwen Wang, Jiyue Zhu

**Affiliations:** Department of Pharmaceutical Sciences, Washington State University College of Pharmacy, PO Box 1495, Spokane, WA 99210, USA; de.cheng@wsu.edu (D.C.); shuwen.wang@wsu.edu (S.W.); jiyue.zhu@wsu.edu (J.Z.)

**Keywords:** telomerase, TERT, transcriptional regulator, chromatin, cancer

## Abstract

Telomerase, regulated primarily by the transcription of its catalytic subunit telomerase reverse transcriptase (TERT), is critical for controlling cell proliferation and tissue homeostasis by maintaining telomere length. Although there is a high conservation between human and mouse TERT genes, the regulation of their transcription is significantly different in these two species. Whereas mTERT expression is widely detected in adult mice, hTERT is expressed at extremely low levels in most adult human tissues and cells. As a result, mice do not exhibit telomere-mediated replicative aging, but telomere shortening is a critical factor of human aging and its stabilization is essential for cancer development in humans. The chromatin environment and epigenetic modifications of the hTERT locus, the binding of transcriptional factors to its promoter, and recruitment of nucleosome modifying complexes all play essential roles in restricting its transcription in different cell types. In this review, we will discuss recent progress in understanding the molecular mechanisms of TERT regulation in human and mouse tissues and cells, and during cancer development.

## 1. Introduction

Telomeres are the protective caps on chromosomal ends that are required for chromosomal stability and cell survival. Telomeres are replenished by telomerase, a ribonucleoprotein complex containing a catalytic subunit (TERT), an RNA template (TERC), and other associated proteins [1]. Telomerase maintains telomere length by adding the hexametric TTAGGG repeats to the 3ʹ end of chromosomes [2]. In most tissues and cells, TERT is the limiting component of the telomerase complex and telomerase activity is associated with the levels of TERT mRNA and protein. In humans, telomeres serve as an aging clock because most somatic cells lack telomerase (i.e., hTERT) expression and their telomeres progressively shorten upon successive cell division. Indeed, studies have shown that telomere shortening is a critical factor of human aging and its stabilization is essential for the development of most human cancers [3]. Human TERT (hTERT) expression increases significantly during tumorigenesis, correlating with the increased proliferative potential of cancer cells [4].

Telomere length regulation and mechanisms of proliferative senescence are not evolutionarily conserved, even among mammals. In a comparative analysis of telomere length and telomerase expression in cells of over 60 mammalian species, Gomes and colleagues concluded that the ancestral mammalian had human-like short telomeres and repressed telomerase expression [5]. Cells in these animals undergo replicative aging, providing a barrier for tumor progression. On the other hand, many other mammals, especially some of the smaller and shorter-lived animals, such as rodents, telomeres become much longer, and telomerase is found in most somatic tissues. These studies provided a conceptual framework for understanding different telomere homeostasis in mammals and identified the need to use appropriate models for studying the role of telomere in human cancer and aging.

Laboratory mice are the most commonly used animal models for human development, aging, and diseases. While telomere length serves as a critical counting mechanism for cellular senescence in human cells, mice do not exhibit telomere-mediated replicative aging. Compared to humans, telomere homeostasis in mice is distinctive in two ways: Laboratory mice express ubiquitous telomerase activities in somatic tissues and possess long heterogeneous telomeres [6,7]. There exist significant differences in telomerase expression between humans and mice [8]. Unlike the hTERT, which is not expressed or expressed at extremely low levels in the most of human somatic tissues and cells, the mouse TERT expression is found in most adult tissues and organs [9,10]. This difference likely results in, or at least contributes to, much longer telomeres (50–100 kb) in laboratorial mice, in comparison to human telomere (5–15 kb) [11]. As a result, telomere length is not apparently a limit to cellular lifespan in mouse cells.

Mouse models of human diseases have become a central part of biomedical research. Laboratory mice provide the most experimentally accessible mammalian models that share genes, organs, and systemic physiology with humans. However, many mouse models do not comprehensively mimic human disease progression, posing challenges in their exploitation to study human diseases. This may have contributed to the high failure rates of human clinical trials, particularly in oncology, predicating the need for improved preclinical data from mouse models [12]. A principal difference between mice and humans relates to a longtime observation that murine fibroblasts grow in culture undergo spontaneous immortalization at a high frequency, owing to their long telomeres and constitutive telomerase expression [13].

## 2. Regulation of TERT Genes

hTERT is a limiting subunit of telomerase in most human tissues. Whereas TERC RNA and other telomerase-associated proteins are expressed in most cell types, hTERT expression is highly regulated and its expression correlated with telomerase activity in many cell types. Studies have indicated that hTERT transcription is the primary step of telomerase regulation [14]. hTERT transcriptional regulation is a complex process that involves both binding of transcription factors, such as Sp1 and c-Myc, to the promoter [15,16,17] and epigenetic mechanisms that regulate chromatin environment of the hTERT locus [17].

**Figure 1:**
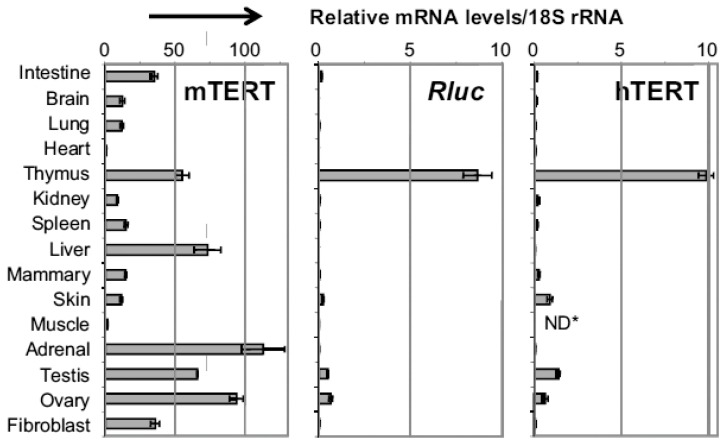
TERT mRNA expression in adult tissues in Tg (hTERT-Rluc) transgenic mice and normal human tissues. mTERT and Rluc (*Renilla* luciferase) mRNA levels in (**left**) and (**middle**) are expressed as percentage of those in pluripotent stem cells. (**right**) shows relative levels of hTERT mRNA. The data were normalized to 18S rRNA. * ND, not determined. The data was originally published in [7].

hTERT expression is stringently regulated in most adult somatic tissues. It was previously shown that the hTERT gene was embedded in a condensed chromatin domain in many somatic cells [18]. This result was correlated well with more recent data from genome-wide chromatin immunoprecipation (ChIP) and high through-put DNA sequencing published at the UCSC genome browser, which shows that this entire region is enriched for repressive histone markers, including H3K9me3 and H3K27me3, but extremely low for H3K36me3, which is correlated with active transcription, in many cells. Consistently, most tissues and organs express no or very low levels of hTERT mRNA (Figure 1). However, there are several exceptions [6,7]. For example, thymus contains the highest level of hTERT mRNA among all human adult tissues, indicating that the hTERT gene is highly expressed in immature T cells. hTERT mRNA is also detectable in ovary, testis, bone marrow, and skin, all of which contain multipotent adult stem cells and/or germline cells. On the other hand, moderate to high levels of mTERT mRNA were found in all tissues and organs, with the exception of heart and skeletal muscle.

**Figure 2:**
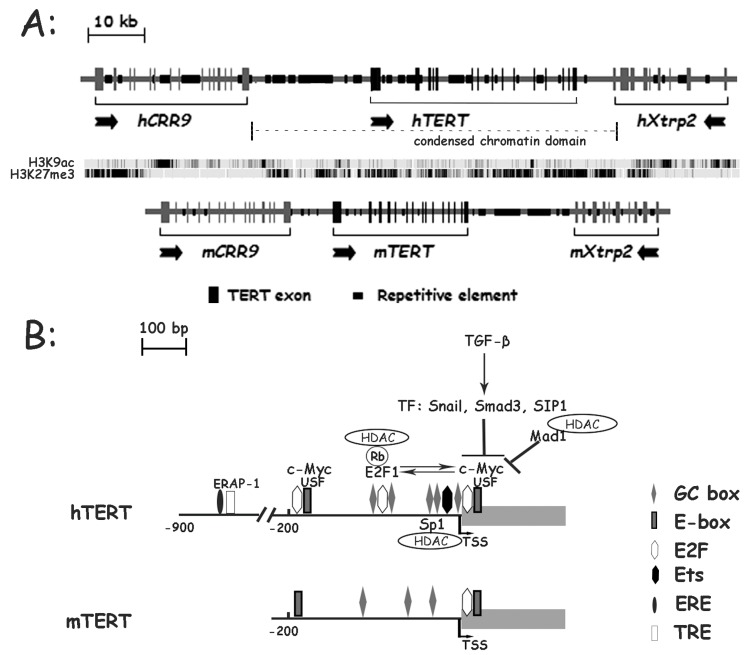
Genomic structures of human and mouse TERT loci. (**A**) Comparison of genomic region containing TERT and neighboring genes in human and mice. The arrows indicate the transcriptional direction of genes. Multiple Alu elements and other human-specific repetitive sequences locate within the 5′ intergenic region and introns of the hTERT locus. hTERT gene region is enriched with repressive histone marker H3K27me3, but extremely low with active marker H3K9ac in K562 cells (date from UCSC website). The dash line indicates a condensed chromatin domain over the hTERT locus. (**B**) Protein factors involved in the regulation of hTERT and mTERT core promoters. The arrows indicate the transcriptional start site (TSS) of TERT gene. ERE: estrogen receptor element; TRE: TPA response element.

One of the bottlenecks in the study of hTERT gene regulation is that the transiently transfected hTERT promoter reporters often do not recapitulate the regulation of endogenous promoter in host cells. For example, transient reporters of the hTERT promoter were similarly active in both telomerase-positive and negative cells [19,20]. This technical issue was overcome by using a chromosomally integrated BAC reporter, which contained a 160-kb human genomic sequence encompassing three consecutive loci, CRR9 (also known as CLPTM1L), hTERT, and Xtrp2 (SLC6A18) (Figure 2A) [21]. The hTERT promoter in this chromosomal reporter was highly active in pluripotent stem cells and silenced upon differentiation into osteogenic cells [21]. A transgenic mouse line Tg (hTERT-Rluc) was created using this BAC reporter (117B23-tR) [7]. In the mouse lines, the level of reporter luciferase mRNA, transcribed from the transgenic hTERT promoter, was the highest in thymus and detectable in testis, ovary, and skin, but negligible in other mouse tissues. This expression profile was identical to that of hTERT mRNA in human tissues, but significantly different from endogenous mTERT expression, which was readily detected in most tissues. Together, these data demonstrated that mouse cells contained the trans-acting factors and epigenetic machineries required for the developmental regulation of hTERT gene.

## 3. Genomic Sequences Underlining the Differential Regulation of hTERT and mTERT Genes

The lack of somatic hTERT expression, compared to those of mTERT, are most likely resulted from its much stronger repression—in human cells, owing to the genomic sequences of the hTERT locus. Indeed, while ectopic hTERT expression is sufficient for the immortalization of many human cell types [22], spontaneous immortalization of human cells occurs extremely rarely, indicating that the repression of hTERT gene is remarkably stringent and stable. On the other hand, it has been known for decades that mouse cells undergo spontaneous immortalization in culture at much higher frequencies than normal human cells. Consequently, mouse cells are much easier to immortalize and transform due to their longer telomere and telomerase expression [9]. Yet, the molecular mechanisms and genomic sequences that underline this stringent repression in human cells remain to be elucidated.

The order of TERT and its neighboring loci, upstream gene CRR9 and downstream Xtrp gene, is the same in human and mice (Figure 2A). The intergenic sequence between CRR9 and hTERT is 23 kb and 6 kb in human and mice, respectively. There are multiple Alu elements and other human-specific repetitive sequences within the 5′ intergenic region and introns of the hTERT locus [23]. In most cells examined, the chromatin of entire hTERT genomic region, from 5′ intergenic region to the downstream Xtrp2 locus, were highly resistant to nuclease-digestion, compared to the corresponding regions of the mTERT locus, indicating that the hTERT gene was embedded in a condensed chromatin domain [18]. There data were consistent with the more recent data of ChIP and high through-put DNA sequencing. hTERT gene region is enriched for repressive histone marker H3K27me3, but extremely low for active marker H3K9ac in K562 cells (Figure 2A) (data from UCSC genome bioinformatics, genome.ucsc.edu). Unlike hTERT promoter, mTERT promoter activity is less dependent on its surrounding chromatin structure. Transfected mTERT promoter was downregulated during cell differentiation [23]. Furthermore, the expression profile of transgenic BAC reporter 117B23-tR, recapitulated the endogenous hTERT gene in human tissues [7], indicating the cis-acting regulatory elements mediated hTERT repression in normal cells. Wilson et al. also demonstrated that the genetic sequence of hTERT locus played a more important role in regulating hTERT expression than the transcription factors and epigenetic modifications [24]. The interspecies differences of genetic sequences and genomic environments around hTERT and mTERT loci are likely critical to the regulatory modes of hTERT and mTERT genes.

Taken together, the interspecies difference of TERT regulation resulting from divergent chromatin environment of hTERT and mTERT loci likely contributes to different telomere length regulation in human and mice. Although primary functions of the TERT protein are evolutionarily conserved, the differential telomerase regulation and telomere length between human and mice have profound impact on the uses of laboratory mice as models of human aging and diseases.

## 4. Transcriptional Regulation of hTERT Promoter

While chromatin environment and epigenetic regulation control hTERT regulation during development and cell differentiation, hTERT transcription is also regulated by binding of transcription factors (TFs) to its promoter. The hTERT promoter contains abundant potential TF binding sites and these sites may play important roles in hTERT transcription in response to changes of physiological conditions and during tumorigenesis. Several sequence-specific transcriptional factors have been identified to bind directly to the hTERT promoter, like c-Myc, USFs, Sp1, Ets, E2F1, AP-1 and ER (Figure 2B) [2,25]. Most of TF binding motifs are conserved between human and mouse TERT core promoters (Figure 2B).

### 4.1. Transcriptional Factors Involved in hTERT Regulation

#### 4.1.1. Myc Family TFs

There are two canonical E-box consensus sites at the hTERT core promoter, located at upstream 165 bp and downstream 45 bp relative to the hTERT transcriptional start site (TSS). These sites are known binding sites for c-Myc super family TF complexes. c-Myc is a proto-oncogene and its overexpression increases telomerase activity by elevating hTERT transcription level in normal human epithelial cells and primary fibroblasts [15]. The repression of hTERT promoter via binding with negative members of Myc super family Max/Mad1 heterodimer was also been reported [26]. Furthermore, hTERT promoter activation was also induced upon c-Myc knockdown through mechanisms independent of E-boxes [27]. Knocking down c-Myc increased the recruitment of multiple TFs, like USF1/2, Sp1 and E2F family proteins, to hTERT promoter, via mechanisms that remained to be elucidated.

Max dimerization protein 1 (Mad1) was identified as a transcriptional repressor during a screening for hTERT regulators [28]. The expression level of Mad1 was very low in most human normal cells, and it was further reduced in some clinical cancer samples [29,30]. Knocking down Mad1 with siRNA induced hTERT transcription in U2OS cells. The Mad1/Max complex binds to E-boxes and represses hTERT transcription while the binding of c-Myc/MAX complex leads to gene activation. The Mad1/Max complex replaced c-Myc/Max at the E-boxes upon cell differentiation [26,28,29]. Further study found that Akt, a serine/threonine kinase, directly bounded to Mad1 and phosphorylated Ser-145 of Mad1, suppressing its interaction with target E-boxes [31]. A dynamic switch between c-Myc and Mad1 to form the heterodimer complex with Max might be one mechanism to control hTERT transcription level in different cell lines.

#### 4.1.2. E2F1

E2F1 promotes cell proliferation and mediates cell apoptosis. There are two non-canonical E2F1 binding sites (E2F) at the hTERT promoter, −174 and −98 (CGCGC) relative to the start of transcription [32]. E2F1 overexpression in SCC25 cells decreased hTERT mRNA level about four-fold [32]. One mutation at −172 (T > C), which located very close to the upstream E2F site, decreased E2F1 binding with hTERT promoter in Huh7 cells [33]. Furthermore, crosstalk between E2F1 and c-Myc might occur because c-Myc overexpression suppressed E2F1 activity [34]. It was proposed that a negative feedback loop, which involved E2F1 and c-Myc, limited c-Myc induction of hTERT expression in normal somatic cells [35,36]. Furthermore, the E2F1-induced negative regulation of c-Myc/hTERT was mediated by miR-17-92 cluster [36]. However, E2F1 played different roles in some cancer cells. Exogenous E2F1 induced hTERT promoter activity in a number of cancer cells, like sarcoma, gliomas, medulloblastoma, and cervical carcinoma cell lines [37,38]. A correlation between E2F1 and hTERT expression was also detected in the malignant gliomas [39], indicating that E2F1 regulated hTERT expression via different mechanisms in different cell types.

#### 4.1.3. TGF-β signaling

Transforming growth factor-β (TGF-β) represses telomerase activity by down-regulating hTERT mRNA level. Snail, one of the downstream targets of TGF-β induction, is highly expressed during embryogenesis and plays a critical role in the stem cell division. Expression of snail was increased in immortalized human cells, in which hTERT transcription was activated [40]. Up-regulated Snail interacted with two E-boxes in hTERT core promoter by competing with c-Myc, which is down-regulated in response to TGF-β induction. Another downstream protein of TGF-β induction, zinc finger transcription factor Smad interacting protein-1 (SIP1), was found to repress hTERT. Depletion of SIP1 with siRNA in U2OS cells did not affect hTERT transcription or translation, but knocking down SIP1 in TGF-β treated MCF7 cells increased hTERT mRNA level [28]. In addition, Smad family member Smad3, also a TGF-β target, repressed hTERT transcription via a c-Myc independent pathway [41].

### 4.2. Histone Acetylation and Deacetylation

Histone acetylation and deacetylation are critical for gene regulation. Trichostatin A (TSA), an inhibitor of classes I and II histone deacetylases (HDACs), increased hTERT mRNA level in normal cells and telomerase-negative immortal cell lines [41,42,43]. Mad1 antagonized c-Myc by binding the E-boxes and recruiting HDACs to the hTERT promoter [44]. Recent study in our lab revealed that the binding of TFs, such as Sp1, to hTERT promoter was essential, but not sufficient, for hTERT transcription. Indeed, HDAC-mediated chromatin remodeling played a central role in repressing hTERT expression in telomerase-negative immortal cells [45].

### 4.3. DNA and Histone Methylation

Genomic sequence around the hTERT promoter is highly GC-rich. Methylation of two CpG islands near the promoter, located at −900 bp and −4000 bp upstream of ATG codon, respectively, was reported to be associated with lower hTERT expression in B-cell lymphocytic leukemia [46]. Aberrant DNA methylation in the promoter was accompanied by alteration of hTERT expression in different cancer cells, like melanoma, esophageal squamous cell carcinoma, and pituitary adenomas [47,48,49]. In addition to DNA methylation, covalent histone modifications are another important factor for gene regulation. Our lab examined the profile of dimethylation of histone H3 lysine 4 (H3K4Me2) and trimethylation of H3 lysine 9 residue (H3K9Me3) in a transgenic hTERT promoter in mouse embryo fibroblasts (MEFs) and induced pluripotent stem cells (iPSCs) derived from them [50]. While H3K4Me2, an active mark of gene expression, was low in MEFs but high in iPSCs, the repressive mark H3K9Me3 decreased progressively in the cells undergoing reprogramming and almost vanished in completely reprogrammed iPSCs [50]. Similar correlation between histone methylations and telomerase activity was also observed in the other studies: H3K9me3 was lower in hTERT positive cells after comparing to the normal cells [51]. Investigation of H3K4Me2 and H3K9Me3 at the hTERT promoter in the cancer cells by combining DNA methylation analysis with ChIP revealed that unmethylated DNA was associated with active chromatin while DNA methylation was preferentially detected around inactive chromatin [52].

### 4.4. hTERT Regulation in Cancer Cells

hTERT expression is induced in cancer cells but absent in most somatic cells. First, transcription activators lead to up-regulation of TERT expression in cancer cells. Increased c-Myc expression in cancer cells resulted in the binding of c-Myc/Max complex to E-boxes at hTERT promoter [28]. Sp1 bonded five consensus GC-boxes at the hTERT core promoter and cooperated with c-Myc to activate hTERT transcription. hTERT transcription was dramatically decreased following the mutation of one GC-box and was eliminated when all five sites were mutated [45,53]. Hormone estrogen was also a regulator of hTERT transcription [54]. Inhibition of estrogen receptor or a mutation of an estrogen response element at the hTERT promoter abolished hTERT reactivation in cancer cells and resulted in cancer cell apoptosis [55,56]. High-risk human papillomavirus type 16 (HPV 16) E6 protein, an E3 ubiquitin ligase, activated hTERT transcription in epithelial cells. The two E-boxes were also essential for the E6-mediated up-regulation of hTERT expression [57], indicating that c-Myc functioned as a downstream effector of E6 protein. In addition, another TF NFX1-123, one variant of NFX1 (nuclear transcription factor, x-box binding 1), was also shown to cooperate with E6 to regulate TERT expression [58].

Second, germline and somatic mutations of the hTERT promoter affect hTERT transcription expression, and impact cancer development. Multiple mutations at the hTERT promoter have been discovered in recent years with the use of next-generation sequencing techniques (Table 1). Two hotspot point mutations, which frequently occurred in urothelial cancer, melanoma, and several other cancers, 124(C/T) and 146(C/T) base pairs upstream of the hTERT ATG codon were found to generate new Ets binding motifs (GGA(A/T)) [59,60,61,62]. hTERT mRNA levels in urothelial cancer cell lines with −124/−146 promoter mutations significantly increased comparing to those without the mutations. As a result, hTERT protein expression level and telomerase activity also were induced [60]. Reversion of −146T to C in urothelial cancer cells decreased the elevated hTERT transcription levels [63]. This result indicated the hTERT reactivation in cancer cell was directly associated with these two mutation sites. The −124/−146 mutant sites recruited more Ets factors to the promoter. Among four Ets factors examined by ChIP, GA-binding protein (GABP) was found to be the critical one that activated hTERT expression in cells containing the −124/−146 mutation [64]. Furthermore, active chromatin mark H3K4me2/3 was detected in the context of hTERT promoter mutant 124(C/T) [65]. Li et al. found 146(C/T), not 124(C/T), recruited Ets1/2 binding by non-canonical NF-κB signaling. 146(C/T) mutation also generated a new half binding site (5′-GGGGG-3′ or 5′-GGAA-3′) for p52. Then, Ets1/2 heterodimer cooperated with p52 to drive hTERT transcription in the presence of 146(C/T) mutation [66]. In addition to regulating hTERT expression and telomerase activity, these two noncoding mutations were found to give rise to monoallelic expression of hTERT [67].

## 5. Single Nucleotide Polymorphisms (SNPs) at the hTERT Locus

Genome-wide association studies revealed that SNP sites at the chromosomal region 5p15.33, where hTERT gene located, were associated with increasing cancer risks [68]. SNPs within the hTERT locus have been found to associate with increased risks of a variety of cancer types. One SNP, rs2736098, which locates in the exon 2 of hTERT, increased the risk for lung cancer in Asian people [69,70,71]. Another SNP in intron 2, rs2736100, was associated with various types of cancer, like colorectal cancer and glioma [72,73,74]. rs2736100 was also found to associate with longer telomeres in peripheral white blood cells in lung cancer patients [75]. Fine mapping on 5p15.33 revealed four independent sites in this locus suspiciously leading to prostate cancer [76]. One variant rs2853669 within a DNase I hypersensitivity region of hTERT promoter linked to multiple cancers [77,78]. This SNP decreased E2F1 binding to its consensus motifs and increased recurrence risks of liver cancer [33]. In addition, SNP rs2736108 in the promoter was predicted to be associated with longer telomeres by analyzing about 480 SNPs at the TERT locus in ovary and breast cancer cell [68].

## 6. Chromosomal Rearrangement at the hTERT Locus

In addition to point mutations, chromosomal rearrangement and amplification involving the hTERT locus have been discovered in immortal cells and cancer cells. In our studies, the chromosomal breakage points have been mapped to the upstream regions of hTERT promoter in telomerase positive immortal cells but not in the telomerase negative cells and their parent cells [89]. The levels of hTERT mRNA were found to be correlated inversely with the distance between the breakage points and the promoter, but not with copy numbers of the hTERT gene. This result suggested that disruption of chromatin environment of the hTERT promoter was a primary cause of hTERT activation. Another study indicated that hTERT, but not its neighboring genes SLC6A18 and SLC6A19, was affected during chromosomal relocalization in neuroblastomas [90]. hTERT rearrangement may be a critical step in cancer development. Thirty-one percent, 12 out of 39 cases, of the high-risk neuroblastoma group contained chromosomal rearrangements at 5p15.33, where hTERT located [91]. These chromosomal rearrangements were associated with upregulated hTERT transcription in neuroblastoma [90]. The ChIP data revealed that active epigenetic marks H3K4me3 and H3K27ac at hTERT promoter were elevated in the hTERT-rearranged tumors comparing to those with no rearrangements [91].

## 7. Conclusions

hTERT expression strictly limits telomerase activation in most of somatic cells, whereas mTERT expression is detectable in most of mouse tissue cells. The interspecies differences between human and mice suggest an improved mouse line, in which both telomerase regulation and telomere length controls are humanized, would considerably benefit the studies of human aging and cancer using mouse models.

## Figures and Tables

**Table 1:** Cancer associated mutations and SNPs at the hTERT promoter.

Site	Position (GRCh37)	A1/A2	Cancer risk	References
124(C/T)	1,295,228	C/T	multiple tumors, like melanoma glioblastomas	[59,79,80,81]
146(C/T)	1,295,250	C/T(CC/TT)		
rs2853669	1,295,349	T/C	glioblastomas	[77,82,83]
			thyroid cancer	[61]
			bladder cancer	[78]
			clear cell renal cell carcinoma	[84]
			hepatocellular carcinoma	[33]
rs2735940	1,296,486	T/C	lung cancer	[85]
			childhood acute lymphoblastic leukemia	[86]
rs2736108	1,297,488	C/T	longer telomere	[68]
rs13174814	1,299,859	G/C	endometrial cancer	[87]
rs421629	1,320,136	C/T	lung cancer	[88]

## References

[1] Venteicher A.S., Abreu E.B., Meng Z., McCann K.E., Terns R.M., Veenstra T.D., Terns M.P., Artandi S.E. (2009). A human telomerase holoenzyme protein required for Cajal body localization and telomere synthesis. Science.

[2] Cong Y.S., Wright W.E., Shay J.W. (2002). Human telomerase and its regulation. Microbiol. Mol. Biol. Rev..

[3] Wright W.E., Shay J.W. (2000). Telomere dynamics in cancer progression and prevention: Fundamental differences in human and mouse telomere biology. Nat. Med..

[4] Akincilar S.C., Unal B., Tergaonkar V. (2016). Reactivation of telomerase in cancer. Cell Mol. Life Sci..

[5] Gomes N.M., Ryder O.A., Houck M.L., Charter S.J., Walker W., Forsyth N.R., Austad S.N., Venditti C., Pagel M., Shay J.W. (2011). Comparative biology of mammalian telomeres: Hypotheses on ancestral states and the roles of telomeres in longevity determination. Aging Cell.

[6] Horikawa I., Chiang Y.J., Patterson T., Feigenbaum L., Leem S.H., Michishita E., Larionov V., Hodes R.J., Barrett J.C. (2005). Differential cis-regulation of human versus mouse TERT gene expression in vivo: Identification of a human-specific repressive element. Proc. Natl. Acad. Sci. USA.

[7] Jia W., Wang S., Horner J.W., Wang N., Wang H., Gunther E.J., DePinho R.A., Zhu J. (2011). A BAC transgenic reporter recapitulates in vivo regulation of human telomerase reverse transcriptase in development and tumorigenesis. FASEB J..

[8] Marti’N-Rivera L., Herrera E.S., Albar J.P., Blasco M.A.A. (1998). Expression of mouse telomerase catalytic subunit in embryos and adult tissues. Proc. Natl. Acad. Sci. USA.

[9] Prowse K.R., Greider C.W. (1995). Developmental and tissue-specific regulation of mouse telomerase and telomere length. Proc. Natl. Acad. Sci. USA.

[10] Greenberg R.A., Allsopp R.C., Chin L., Morin G.B., DePinho R.A. (1998). Expression of mouse telomerase reverse transcriptase during development, differentiation and proliferation. Oncogene.

[11] Kipling D., Cooke H.J. (1990). Hypervariable ultra-long telomeres in mice. Nature.

[12] Singh M., Murriel C.L., Johnson L. (2012). Genetically engineered mouse models: Closing the gap between preclinical data and trial outcomes. Cancer Res..

[13] Rangarajan A., Weinberg R.A. (2003). Opinion: Comparative biology of mouse versus human cells: Modelling human cancer in mice. Nat. Rev. Cancer.

[14] Aisner D.L., Wright W.E., Shay J.W. (2002). Telomerase regulation: Not just flipping the switch. Curr. Opin. Genet. Dev..

[15] Takakura M., Kyo S., Kanaya T., Hirano H., Takeda J., Yutsudo M., Inoue M. (1999). Cloning of human telomerase catalytic subunit (hTERT) gene promoter and identification of proximal core promoter sequences essential for transcriptional activation in immortalized and cancer cells. Cancer Res..

[16] Wu K.J., Grandori C., Amacker M., Simon-Vermot N., Polack A., Lingner J., Dalla-Favera R. (1999). Direct activation of TERT transcription by c-MYC. Nat. Genet..

[17] Zhu J., Zhao Y., Wang S. (2010). Chromatin and epigenetic regulation of the telomerase reverse transcriptase gene. Protein Cell.

[18] Wang S., Zhu J. (2004). The hTERT gene is embedded in a nuclease-resistant chromatin domain. J. Biol. Chem..

[19] Ducrest A.L., Amacker M., Mathieu Y.D., Cuthbert A.P., Trott D.A., Newbold R.F., Nabholz M., Lingner J. (2001). Regulation of human telomerase activity: Repression by normal chromosome 3 abolishes nuclear telomerase reverse transcriptase transcripts but does not affect c-Myc activity. Cancer Res..

[20] Wang S., Zhu J. (2003). Evidence for a relief of repression mechanism for activation of the human telomerase reverse transcriptase promoter. J. Biol. Chem..

[21] Wang S., Hu C., Zhu J. (2007). Transcriptional silencing of a novel hTERT reporter locus during in vitro differentiation of mouse embryonic stem cells. Mol. Biol. Cell..

[22] Bodnar A.G., Ouellette M., Frolkis M., Holt S.E., Chiu C.P., Morin G.B., Harley C.B., Shay J.W., Lichtsteiner S., Wright W.E. (1998). Extension of life-span by introduction of telomerase into normal human cells. Science.

[23] Wang S., Zhao Y., Hu C., Zhu J. (2009). Differential repression of human and mouse TERT genes during cell differentiation. Nucleic Acids Res..

[24] Wilson M.D., Barbosa-Morais N.L., Schmidt D., Conboy C.M., Vanes L., Tybulewicz V.L., Fisher E.M., Tavare S., Odom D.T. (2008). Species-specific transcription in mice carrying human chromosome 21. Science.

[25] Horikawa I., Barrett J.C. (2003). Transcriptional regulation of the telomerase hTERT gene as a target for cellular and viral oncogenic mechanisms. Carcinogenesis.

[26] Xu D., Popov N., Hou M., Wang Q., Bjorkholm M., Gruber A., Menkel A.R., Henriksson M. (2001). Switch from Myc/Max to Mad1/Max binding and decrease in histone acetylation at the telomerase reverse transcriptase promoter during differentiation of HL60 cells. Proc. Natl. Acad. Sci. USA.

[27] Zhao Y., Cheng D., Wang S., Zhu J. (2014). Dual roles of c-Myc in the regulation of hTERT gene. Nucleic Acids Res..

[28] Lin S.Y., Elledge S.J. (2003). Multiple tumor suppressor pathways negatively regulate telomerase. Cell.

[29] Gunes C., Lichtsteiner S., Vasserot A.P., Englert C. (2000). Expression of the hTERT gene is regulated at the level of transcriptional initiation and repressed by Mad1. Cancer Res..

[30] Han S., Park K., Kim H.Y., Lee M.S., Kim H.J., Kim Y.D. (1999). Expression of Mad1 protein inhibits proliferation of cancer cells and inversely correlated with Myc protein expression in primary gastric cancer. Oncol. Rep..

[31] Chou C.K., Lee D.F., Sun H.L., Li L.Y., Lin C.Y., Huang W.C., Hsu J.M., Kuo H.P., Yamaguchi H., Wang Y.N. (2009). The suppression of MAD1 by AKT-mediated phosphorylation activates MAD1 target genes transcription. Mol. Carcinog..

[32] Crowe D.L., Nguyen D.C., Tsang K.J., Kyo S. (2001). E2F-1 represses transcription of the human telomerase reverse transcriptase gene. Nucleic Acids Res..

[33] Ko E., Seo H.W., Jung E.S., Kim B.H., Jung G. (2016). The TERT promoter SNP rs2853669 decreases E2F1 transcription factor binding and increases mortality and recurrence risks in liver cancer. Oncotarget.

[34] Coller H.A., Forman J.J., Legesse-Miller A. (2007). “Myc’ed Messages”: Myc Induces Transcription of E2F1 while Inhibiting Its Translation via a microRNA Polycistron. PLoS Genet..

[35] Zhang Y., Chen L., Yang S., Fang D. (2012). E2F1: A potential negative regulator of hTERT transcription in normal cells upon activation of oncogenic c-Myc. Med. Sci. Monit..

[36] Zhang Y., Zhang A., Shen C., Zhang B., Rao Z., Wang R., Yang S., Ning S., Mao G., Fang D. (2014). E2F1 acts as a negative feedback regulator of c-Mycinduced hTERT transcription during tumorigenesis. Oncol. Rep..

[37] Alonso M.M., Fueyo J., Yung W.K., Gomez-Manzano C. (2006). E2F1 and telomerase: alliance in the dark side. Cell Cycle.

[38] Alonso M.M., Fueyo J., Shay J.W., Aldape K.D., Jiang H., Lee O.H., Johnson D.G., Xu J., Kondo Y., Kanzawa T. (2005). Expression of transcription factor E2F1 and telomerase in glioblastomas: Mechanistic linkage and prognostic significance. J. Natl. Cancer Inst..

[39] Alonso M.M., Fueyo J., Gomez-Manzano C. (2011). Malignant Gliomas: Role of E2F1 Transcription Factor. Tumors Cent. Nervous Syst..

[40] Yoo Y.S., Park S., Gwak J., Ju B.G., Oh S. (2015). Involvement of transcription repressor Snail in the regulation of human telomerase reverse transcriptase (hTERT) by transforming growth factor-beta. Biochem. Biophys. Res. Commun..

[41] Kyo S., Takakura M., Fujiwara T., Inoue M. (2008). Understanding and exploiting hTERT promoter regulation for diagnosis and treatment of human cancers. Cancer Sci..

[42] Wang S., Hu C., Zhu J. (2010). Distinct and temporal roles of nucleosomal remodeling and histone deacetylation in the repression of the hTERT gene. Mol. Biol. Cell..

[43] Daniel M., Peek G.W., Tollefsbol T.O. (2012). Regulation of the human catalytic subunit of telomerase (hTERT). Gene.

[44] Cong Y.S., Bacchetti S. (2000). Histone deacetylation is involved in the transcriptional repression of hTERT in normal human cells. J. Biol. Chem..

[45] Cheng D., Zhao Y., Wang S., Jia W., Kang J., Zhu J. (2015). Human Telomerase Reverse Transcriptase (hTERT) Transcription Requires Sp1/Sp3 Binding to the Promoter and a Permissive Chromatin Environment. J. Biol. Chem..

[46] Bechter O.E., Eisterer W., Dlaska M., Kühr T., Thaler J. (2002). CpG island methylation of the hTERT promoter is associated with lower telomerase activity in B-cell lymphocytic leukemia. Exp. Hematol..

[47] Fan Y., Lee S., Wu G., Easton J., Yergeau D., Dummer R., Vogel P., Kirkwood J.M., Barnhill R.L., Pappo A. (2016). Telomerase Expression by Aberrant Methylation of the TERT Promoter in Melanoma Arising in Giant Congenital Nevi. J. Investig. Dermatol..

[48] Deng J., Zhou D., Zhang J., Chen Y., Wang C., Liu Y., Zhao K. (2015). Aberrant methylation of the TERT promoter in esophageal squamous cell carcinoma. Cancer Genet..

[49] Kochling M., Ewelt C., Furtjes G., Peetz-Dienhart S., Koos B., Hasselblatt M., Paulus W., Stummer W., Brokinkel B. (2016). hTERT promoter methylation in pituitary adenomas. Brain Tumor Pathol..

[50] Mathew R., Jia W., Sharma A., Zhao Y., Clarke L.E., Cheng X., Wang H., Salli U., Vrana K.E., Robertson G.P. (2010). Robust activation of the human but not mouse telomerase gene during the induction of pluripotency. FASEB J..

[51] Atkinson S.P., Hoare S.F., Glasspool R.M., Keith W.N. (2005). Lack of telomerase gene expression in alternative lengthening of telomere cells is associated with chromatin remodeling of the hTR and hTERT gene promoters. Cancer Res..

[52] Zinn R.L., Pruitt K., Eguchi S., Baylin S.B., Herman J.G. (2007). hTERT is expressed in cancer cell lines despite promoter DNA methylation by preservation of unmethylated DNA and active chromatin around the transcription start site. Cancer Res..

[53] Kyo S., Takakura M., Taira T., Kanaya T., Itoh H., Yutsudo M., Ariga H., Inoue M. (2000). Sp1 cooperates with c-Myc to activate transcription of the human telomerase reverse transcriptase gene (hTERT). Nucleic Acids Res..

[54] Kyo S., Takakura M., Kanaya T., Zhuo W., Fujimoto K., Nishio Y., Orimo A., Inoue M. (1999). Estrogen activates telomerase. Cancer Res..

[55] Kondoh K., Tsuji N., Asanuma K., Kobayashi D., Watanabe N. (2007). Inhibition of estrogen receptor beta-mediated human telomerase reverse transcriptase gene transcription via the suppression of mitogen-activated protein kinase signaling plays an important role in 15-deoxy-Delta(12,14)-prostaglandin J(2)-induced apoptosis in cancer cells. Exp. Cell. Res..

[56] Grasselli A., Nanni S., Colussi C., Aiello A., Benvenuti V., Ragone G., Moretti F., Sacchi A., Bacchetti S., Gaetano C. (2008). Estrogen receptor-alpha and endothelial nitric oxide synthase nuclear complex regulates transcription of human telomerase. Circ. Res..

[57] Veldman T., Liu X., Yuan H., Schlegel R. (2003). Human papillomavirus E6 and Myc proteins associate in vivo and bind to and cooperatively activate the telomerase reverse transcriptase promoter. Proc. Natl. Acad. Sci. USA.

[58] Katzenellenbogen R.A., Vliet-Gregg P., Xu M., Galloway D.A. (2009). NFX1-123 increases hTERT expression and telomerase activity posttranscriptionally in human papillomavirus type 16 E6 keratinocytes. J. Virol..

[59] Horn S., Figl A., Rachakonda P.S., Fischer C., Sucker A., Gast A., Kadel S., Moll I., Nagore E., Hemminki K. (2013). TERT promoter mutations in familial and sporadic melanoma. Science.

[60] Borah S., Xi L., Zaug A.J., Powell N.M., Dancik G., Cohen S.B., Costello J.C., Theodorescu D., Cech T.R. (2015). TERT promoter mutations and telomerase reactivation in urothelial cancer. Science.

[61] Muzza M., Colombo C., Rossi S., Tosi D., Cirello V., Perrino M., de Leo S., Magnani E., Pignatti E., Vigo B. (2015). Telomerase in differentiated thyroid cancer: Promoter mutations, expression and localization. Mol. Cell Endocrinol..

[62] Zagni C., Chiacchi U., Rescifina A. (2013). Histone Methyltransferase Inhibitors: Novel Epigenetic Agents for Cancer Treatment. Curr. Med. Chem..

[63] Xi L., Schmidt J.C., Zaug A.J., Ascarrunz D.R., Cech T.R. (2015). A novel two-step genome editing strategy with CRISPR-Cas9 provides new insights into telomerase action and TERT gene expression. Genome Biol..

[64] Bell R.J., Rube H.T., Kreig A., Mancini A., Fouse S.D., Nagarajan R.P., Choi S., Hong C., He D., Pekmezci M. (2015). Cancer. The transcription factor GABP selectively binds and activates the mutant TERT promoter in cancer. Science.

[65] Stern J.L., Theodorescu D., Vogelstein B., Papadopoulos N., Cech T.R. (2015). Mutation of the TERT promoter, switch to active chromatin, and monoallelic TERT expression in multiple cancers. Genes Dev..

[66] Li Y., Zhou Q.L., Sun W., Chandrasekharan P., Cheng H.S., Ying Z., Lakshmanan M., Raju A., Tenen D.G., Cheng S.Y. (2015). Non-canonical NF-kappaB signalling and ETS1/2 cooperatively drive C250T mutant TERT promoter activation. Nat. Cell Biol..

[67] Huang F.W., Bielski C.M., Rinne M.L., Hahn W.C., Sellers W.R., Stegmeier F., Garraway L.A., Kryukov G.V. (2015). TERT promoter mutations and monoallelic activation of TERT in cancer. Oncogenesis.

[68] Bojesen S.E., Pooley K.A., Johnatty S.E., Beesley J., Michailidou K., Tyrer J.P., Edwards S.L., Pickett H.A., Shen H.C., Smart C.E. (2013). Multiple independent variants at the TERT locus are associated with telomere length and risks of breast and ovarian cancer. Nat. Genet..

[69] Wu H., Qiao N., Wang Y., Jiang M., Wang S., Wang C., Hu L. (2013). Association between the telomerase reverse transcriptase (TERT) rs2736098 polymorphism and cancer risk: evidence from a case-control study of non-small-cell lung cancer and a meta-analysis. PLoS ONE.

[70] Zhang C., Tian Y.P., Wang Y., Guo F.H., Qin J.F., Ni H. (2013). hTERT rs2736098 genetic variants and susceptibility of hepatocellular carcinoma in the Chinese population: A case-control study. Hepatobiliary Pancreat Dis. Int..

[71] Zhang X.J., Xu Z., Gong Y.L., Tang C.J., Chen J.F. (2012). Association of TERT rs2736098 polymorphism with cancer risk: A meta-analysis. Asian Pac. J. Cancer Prev..

[72] Zhou P., Wei L., Xia X., Shao N., Qian X., Yang Y. (2014). Association between telomerase reverse transcriptase rs2736100 polymorphism and risk of glioma. J. Surg. Res..

[73] Wei R., Cao L., Pu H., Wang H., Zheng Y., Niu X., Weng X., Zhang H., Favus M.J., Zhang L. (2015). TERT Polymorphism rs2736100-C Is Associated with EGFR Mutation-Positive Non-Small Cell Lung Cancer. Clin. Cancer Res..

[74] Kinnersley B., Migliorini G., Broderick P., Whiffin N., Dobbins S.E., Casey G., Hopper J., Sieber O., Lipton L., Kerr D.J. (2012). The TERT variant rs2736100 is associated with colorectal cancer risk. Br. J. Cancer.

[75] Lan Q., Cawthon R., Gao Y., Hu W., Hosgood H.D., Barone-Adesi F., Ji B.T., Bassig B., Chow W.H., Shu X. (2013). Longer telomere length in peripheral white blood cells is associated with risk of lung cancer and the rs2736100 (CLPTM1L-TERT) polymorphism in a prospective cohort study among women in China. PLoS ONE.

[76] Kote-Jarai Z., Saunders E.J., Leongamornlert D.A., Tymrakiewicz M., Dadaev T., Jugurnauth-Little S., Ross-Adams H., Al Olama A.A., Benlloch S., Halim S. (2013). Fine-mapping identifies multiple prostate cancer risk loci at 5p15, one of which associates with TERT expression. Hum. Mol. Genet..

[77] Batista R., Cruvinel-Carloni A., Vinagre J., Peixoto J., Catarino T.A., Campanella N.C., Menezes W., Becker A.P., de Almeida G.C., Matsushita M.M. (2016). The prognostic impact of TERT promoter mutations in glioblastomas is modified by the rs2853669 single nucleotide polymorphism. Int. J. Cancer.

[78] Rachakonda P.S., Hosen I., de Verdier P.J., Fallah M., Heidenreich B., Ryk C., Wiklund N.P., Steineck G., Schadendorf D., Hemminki K. (2013). TERT promoter mutations in bladder cancer affect patient survival and disease recurrence through modification by a common polymorphism. Proc. Natl. Acad. Sci. USA.

[79] Huang F.W., Hodis E., Xu M.J., Kryukov G.V., Chin L., Garraway L.A. (2013). Highly Recurrent TERT Promoter Mutations in Human Melanoma. Science.

[80] Simon M., Hosen I., Gousias K., Rachakonda S., Heidenreich B., Gessi M., Schramm J., Hemminki K., Waha A., Kumar R. (2015). TERT promoter mutations: A novel independent prognostic factor in primary glioblastomas. Neuro-Oncol..

[81] Vinagre J., Almeida A., Populo H., Batista R., Lyra J., Pinto V., Coelho R., Celestino R., Prazeres H., Lima L. (2013). Frequency of TERT promoter mutations in human cancers. Nat. Commun..

[82] Mosrati M.A., Malmström A., Lysiak M., Krysztofiak A., Hallbeck M., Milos P., Hallbeck A.-L. (2015). TERT promoter mutations and polymorphisms as prognostic factors in primary glioblastoma. Oncotarget.

[83] Nencha U., Rahimian A., Giry M., Sechi A., Mokhtari K., Polivka M., Schmitt Y., di Stefano A.L., Alentorn A., Labussiere M. (2016). TERT promoter mutations and rs2853669 polymorphism: Prognostic impact and interactions with common alterations in glioblastomas. J. Neurooncol..

[84] Hosen I., Rachakonda P.S., Heidenreich B., Sitaram R.T., Ljungberg B., Roos G., Hemminki K., Kumar R. (2015). TERT promoter mutations in clear cell renal cell carcinoma. Int. J. Cancer.

[85] Catarino R., Araujo A., Coelho A., Gomes M., Nogueira A., Lopes C., Medeiros R.M. (2010). Prognostic significance of telomerase polymorphism in non-small cell lung cancer. Clin. Cancer Res..

[86] Sheng X., Tong N., Tao G., Luo D., Wang M., Fang Y., Li J., Xu M., Zhang Z., Wu D. (2013). TERT polymorphisms modify the risk of acute lymphoblastic leukemia in Chinese children. Carcinogenesis.

[87] Carvajal-Carmona L.G., O’Mara T.A., Painter J.N., Lose F.A., Dennis J., Michailidou K., Tyrer J.P., Ahmed S., Ferguson K., Healey C.S. (2015). Candidate locus analysis of the TERT-CLPTM1L cancer risk region on chromosome 5p15 identifies multiple independent variants associated with endometrial cancer risk. Hum. Genet..

[88] Scherf D.B., Sarkisyan N., Jacobsson H., Claus R., Bermejo J.L., Peil B., Gu L., Muley T., Meister M., Dienemann H. (2013). Epigenetic screen identifies genotype-specific promoter DNA methylation and oncogenic potential of CHRNB4. Oncogene.

[89] Zhao Y., Wang S., Popova E.Y., Grigoryev S.A., Zhu J. (2009). Rearrangement of upstream sequences of the hTERT gene during cellular immortalization. Genes Chromosomes Cancer.

[90] Cobrinik D., Ostrovnaya I., Hassimi M., Tickoo S.K., Cheung I.Y., Cheung N.K. (2013). Recurrent pre-existing and acquired DNA copy number alterations, including focal TERT gains, in neuroblastoma central nervous system metastases. Genes Chromosomes Cancer.

[91] Peifer M., Hertwig F., Roels F., Dreidax D., Gartlgruber M., Menon R., Kramer A., Roncaioli J.L., Sand F., Heuckmann J.M. (2015). Telomerase activation by genomic rearrangements in high-risk neuroblastoma. Nature.

